# Nonlinear Dynamic Behavior of a Bi-Axial Torsional MEMS Mirror with Sidewall Electrodes

**DOI:** 10.3390/mi7030042

**Published:** 2016-03-18

**Authors:** Mehmet Ozdogan, Shahrzad Towfighian

**Affiliations:** 1 Mechanical Engineering Department, State University of New York at Binghamton, Binghamton, NY 13902, USA; mozdoga1@binghamton.edu; 2 Mechanical Engineering Department, Turkish Military Academy, Ankara 06420, Turkey

**Keywords:** MEMS micro-mirror, sidewall electrodes, bi-axial mirror, softening behavior, nonlinear dynamics, secondary resonances

## Abstract

Nonlinear dynamic responses of a Micro-Electro-Mechanical Systems (MEMS) mirror with sidewall electrodes are presented that are in close agreement with previously-reported experimental data. An analysis of frequency responses reveals softening behavior, and secondary resonances originated from the dominant quadratic nonlinearity. The quadratic nonlinearity is an electromechanical coupling effect caused by the electrostatic force. This effect is reflected in our mathematical model used to simulate the dynamic response of the micro-mirror. The effects of increased forcing and decreased damping on the frequency response are investigated as the mirrors are mostly used in vacuum packages. The results can predict MEMS mirror behaviors in optical devices better than previously-reported models.

## 1. Introduction

Micro-electro-mechanical systems (MEMS) are becoming mainstream using recent micro-fabrication methods (e.g., silicon bulk micro-machining and surface micro-machining [1]). Biaxial micro-mirrors are among MEMS devices adopted in numerous areas, such as laser imaging, image digitizing, projection displays [2] and medical applications [3], e.g., endoscopy and tomography. Features to be mostly considered for micro-mirrors include simple fabrication processes, low driving voltages, large tilt angles and linearized angular scans [4]. Actuation mechanisms used for micro-mirrors include electrostatic [5,6], piezoelectric [7,8], electrothermal [9] and electromagnetic [10,11] actuators. Electrostatic actuators are the most popular because of their easy fabrication, low power consumption and high driving speed [12]. However, they require large operating voltages to create large tilting angles. Furthermore, for the MEMS devices that use parallel plate capacitors, pull-in instability is an important concern [13]. Electrothermal actuation provides large deflection angles at low voltages at the cost of low driving speed, large energy consumption and possible changes in optical behavior caused by fluctuating temperatures [14]. Piezoelectric actuators are faster and use lower driving voltages compared to electrothermal devices [7]. However, they are difficult to fabricate. Electromagnetic actuators can achieve large mechanical tilting angles [10], but at the price of a larger size. Among these actuation types, the electrostatic actuator is preferred due to the ease of fabrication in large volumes. Common electrostatic actuator sub-types are the comb-drive and parallel plate. Parallel plate actuators are preferred because of their smaller size compared to comb-drive actuators, but they suffer from pull-in voltage. Because of the nature of electrostatic force, electrostatic MEMS actuators have strong nonlinear behavior, which has been investigated during the past decade [15,16,17,18].

A mirror actuated by sidewall and bottom electrodes was presented by Bai *et al.* [19]. The sidewalls added forces needed to increase the mirror’s angles of rotation about two perpendicular axes. Static and dynamic models of the mirror were introduced by Bai *et al.* [19]; however, these models were unable to predict the nonlinear dynamic responses they reported experimentally. A comprehensive model that accurately predicts a MEMS mirror’s response to voltage excitation would be useful in characterizing optical scanning applications. In our earlier study [20], we presented static modeling of the mirror with sidewall electrodes.

The contribution of this study is to improve the model of the MEMS mirror with sidewalls by accounting for the increased forces and torques on the mirror as the rotation angle increases. Thorough modeling of the electrostatic field more accurately predicts the nonlinear static response and, for the first time, results in close agreement of the simulated nonlinear dynamic response with the experimental results. The analytical model now explains the dominant quadratic nonlinearity in the system, and we verified the prediction with evidence from secondary resonances in the experiments.

The structure of the paper is as follows: the static modeling and simulation of the MEMS mirror under electrostatic forces from sidewall and bottom electrodes is presented. Dynamic modeling and simulation that include the effect of forcing on the frequency responses are presented next in this paper. Finally, secondary resonances evident in experimental data are explained using a mathematical analysis of the equation of motion.

## 2. Static Simulation

### 2.1. Operation Principles

**Figure 1:**
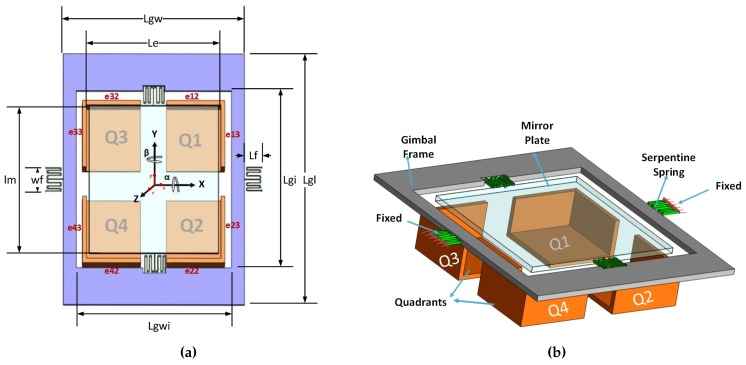
(**a**) Parametric dimensions of the structure; (**b**) 3D model of the mirror elements and quadrants (Adapted from [19]).

**Figure 2:**
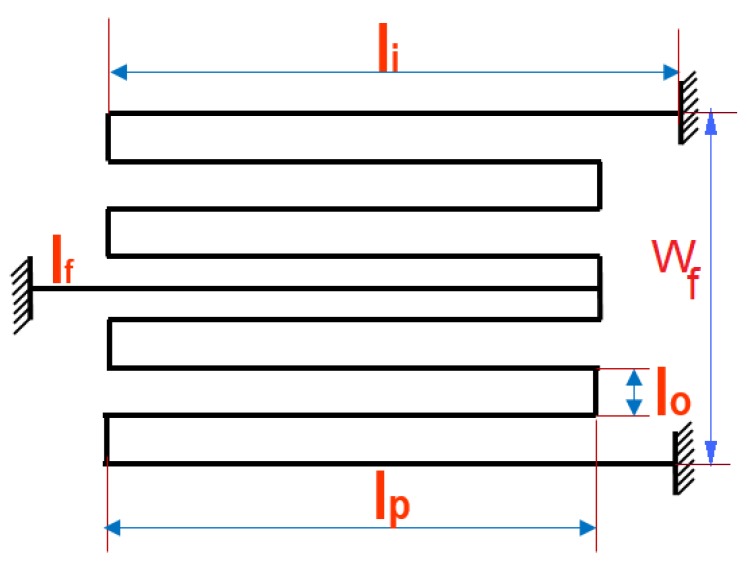
Serpentine spring dimensions (μm): $l_{o}=10$, $l_{p}=110$, $l_{f}=120$, $l_{i}=120$, $w_{f}=160$, w=3μm (width), and $t_{b}=12\;$μm (thickness).

The micro-mirror structure consists of five main components shown in Figure 1: mirror plate, gimbal frame, sidewall electrodes, bottom electrode and serpentine spring (torsion bar). The mirror is made of silicon and is connected to the ground, and a voltage is applied to the sidewall and bottom electrodes. Different voltage potentials between the mirror plate and the sidewall and bottom electrodes create electrostatic torques that rotate the mirror. A schematic of the micro-mirror is shown in Figure 1. The micro-mirror is suspended by the double-gimbal structure, which has two pairs of serpentine springs (Figure 2) for two degree-of-freedom (DOF) scans: the *α* and *β* scan, which are about the *X*-axis and *Y*-axis, respectively. The mirror actuators consist of four equivalent electrode quadrants (also sidewall electrodes) numbered as in Figure 1. The parameters of the micro-mirror are listed in [19]. To operate the mirror, the two rotation angles are generated independently. For the rotation about the *Y*-axis (*β*),

(1) $$\begin{array}{cc} V_{1} & =V_{2}=V_{bias}+V_{dif} \end{array}$$

(2) $$\begin{array}{cc} V_{3} & =V_{4}=V_{bias} \end{array}$$

where $V_{bias}$ is the bias voltage, $V_{dif}$ is the differential voltage, and $V_{1...4}$ is the voltage on the sidewall and bottom electrode of each quadrant. For the rotation about the *X*-axis (*α*),

(3) $$\begin{array}{cc} V_{1} & =V_{3}=V_{bias} \end{array}$$

(4) $$\begin{array}{cc} V_{2} & =V_{4}=V_{bias}+V_{dif} \end{array}$$

In other words, when quadrants Q1 and Q2 have equal and larger voltage compared to Q3 and Q4, the rotation will be pure *β*. Similarly, if quadrants Q2 and Q4 have equal and larger voltage compared to Q1 and Q3, the rotation will be pure *α*.

### 2.2. System Model

#### 2.2.1. Electrostatic Forces and Torques

The global coordinate XYZ is fixed at the center of the initial position of the mirror. The body fixed coordinate XYZ is fixed at the center of the mirror plate and rotates with the micro-mirror. Rotations about the *X* and *Y* axes are independent. Using the defined coordinate systems, expressions of the forces and torques on the mirror from sidewall electrodes, gimbal frame and bottom electrodes are obtained in this section for angle *α*. The derivations for angle *β* may be obtained by following the same method.

##### Sidewall Electrodes

**Figure 3:**
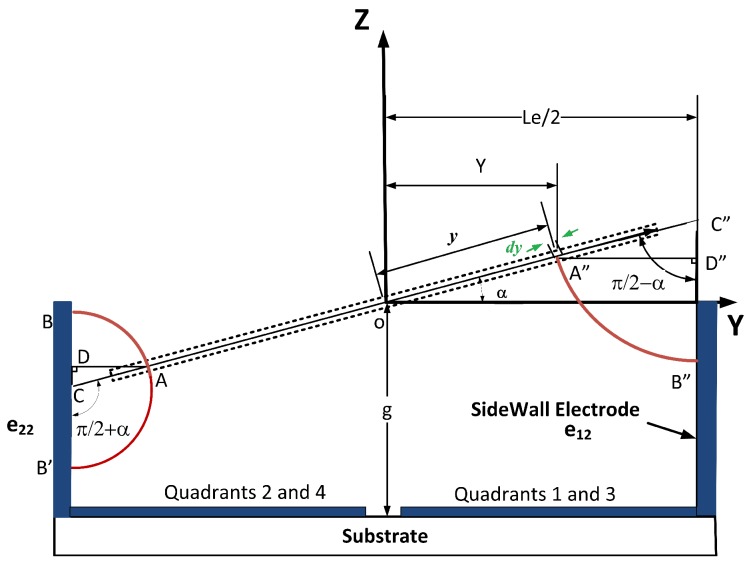
The mirror rotated about the *X*-axis by *α* and Sidewalls 12 and 22 projected on the YZ plane.

Figure 3 shows the projection of the mirror plate on the YZ plane. The center of the mirror plate passes through the center of the global coordinate. The figure shows the mirror rotation about the *X*-axis when Quadrants 2 and 4 have equal and higher voltage than Quadrants 1 and 3. In Figure 3, as the torques about the *Y*-axis balance each other, there will not be any rotation about the *Y*-axis (β=0). The model for electrostatic forces and torques here follows the procedure explained in Bai *et al.* [19], which considers the electrostatic forces from Sidewall 22 and 12 on the bottom surface of the mirror. We simplified this approach based on the operation principle in the previous section (that the two angles are operated independently). We also considered the electrostatic forces from the sidewall electrode on the top surface of the mirror as they become significant in large rotation angles. To incorporate this effect at large angles, the integral boundaries are defined to depend on the rotation angles obtained in the previous voltage step of the simulations. For an element of d*y* on the top layer of the mirror plate, the electrostatic flux [21] from Sidewall 22 on the top surface of the mirror is written as:

(5) $$\begin{array}{c} E_{e22t}=\frac{V_{2}}{\overset{⌢}{AB}} \end{array}$$

where $V_{2}$ is the voltage applied on Quadrant 2 and:

(6) $$\begin{array}{cc} \overset{⌢}{AB}=AC\cdot \left(\pi /2-\alpha \right) & =\frac{AD}{\sin(\pi /2-\alpha )}\left(\pi /2-\alpha \right)\\ & =\left(\frac{L_{e}}{2}-Y\right)\frac{\pi /2-\alpha }{\sin(\pi /2-\alpha )} \end{array}$$

where Le is the bottom electrode edge width and *α* is the rotation angle about the *X*-axis. Substituting Y=y·cosα for a body fixed Point A, the flux Equation (5) is written as:

(7) $$E_{e22t}=\frac{V_{2}\sin\left(\pi /2-\alpha \right)}{\left(\frac{L_{e}}{2}-y\cdot \cos\alpha \right)\left(\pi /2-\alpha \right)}$$

The electrostatic force per unit area [21] is:

(8) $$P=\frac{\varepsilon_{0}E^{2}}{2}$$

where $\varepsilon_{0}$ is air permittivity. Based on Equation (8), the electrostatic force on an element with dimensions of d*x* and d*y* is:

(9) $$dF=\frac{\varepsilon_{0}E^{2}\;dx\;dy}{2}$$

The force in the Z direction from the sidewall e22 is then found from integration:

(10) $$Fz_{e22t}=\frac{\varepsilon_{0}\cdot \sin^{2}\left(\pi /2-\alpha \right)\cdot V_{2}^{2}}{2{\left(\pi /2-\alpha \right)}^{2}}\cdot \int_{y_{1}}^{y_{2}}\int_{x_{1}}^{x_{2}}{\left\{\frac{1}{\frac{L_{e}}{2}-y\cdot \cos\alpha }\right\}}^{2}\;dx\;dy$$

For the integral boundaries, see Appendix. From the force equation, one can find the torque about the *X*-axis from the sidewall e22 on the top surface of the mirror plate.

(11) $$Tx_{e22t}=-\frac{\varepsilon_{0}\cdot \sin^{2}\left(\pi /2-\alpha \right)\cdot V_{2}^{2}}{2{\left(\pi /2-\alpha \right)}^{2}}\cdot \int_{y_{1}}^{y_{2}}\int_{x_{1}}^{x_{2}}{\left\{\frac{1}{\frac{L_{e}}{2}-y\cdot \cos\alpha }\right\}}^{2}\cdot y\;dx\;dy$$

For an element of dy at the bottom surface of the mirror plate, similarly, the electrostatic force in the Z direction from the sidewall e22 on the bottom part of the mirror is:

(12) $$Fz_{e22b}=-\frac{\varepsilon_{0}\cdot \sin^{2}\left(\pi /2-\alpha \right)\cdot V_{2}^{2}}{2{\left(\pi /2+\alpha \right)}^{2}}\cdot \int_{y_{3}}^{y_{4}}\int_{x_{3}}^{x_{4}}{\left\{\frac{1}{\frac{L_{e}}{2}-y\cdot \cos\alpha }\right\}}^{2}\;dx\;dy$$

The torque about the *X*-axis from the sidewall e22 at the bottom of the mirror plate is then:

(13) $$Tx_{e22b}=\frac{\varepsilon_{0}\cdot \sin^{2}\left(\pi /2-\alpha \right)\cdot V_{2}^{2}}{2{\left(\pi /2+\alpha \right)}^{2}}\cdot \int_{y_{3}}^{y_{4}}\int_{x_{3}}^{x_{4}}{\left\{\frac{1}{\frac{L_{e}}{2}-y\cdot \cos\alpha }\right\}}^{2}\cdot y\;dx\;dy$$

In a similar approach, the force and torque on the mirror plate from Sidewall 12 can be derived:

(14) $$\begin{array}{c} Fz_{e12}=-\frac{\varepsilon_{0}\cdot \sin^{2}\left(\pi /2-\alpha \right)\cdot V_{1}^{2}}{2{\left(\pi /2-\alpha \right)}^{2}}\cdot \int_{y_{3}}^{y_{4}}\int_{x_{3}}^{x_{4}}{\left\{\frac{1}{\frac{L_{e}}{2}-y\cdot \cos\alpha }\right\}}^{2}\;dx\;dy\\ Tx_{e12}=-\frac{\varepsilon_{0}\cdot \sin^{2}\left(\pi /2-\alpha \right)\cdot V_{1}^{2}}{2{\left(\pi /2-\alpha \right)}^{2}}\cdot \int_{y_{3}}^{y_{4}}\int_{x_{3}}^{x_{4}}{\left\{\frac{1}{\frac{L_{e}}{2}-y\cdot \cos\alpha }\right\}}^{2}\cdot y\;dx\;dy \end{array}$$

**Figure 4:**
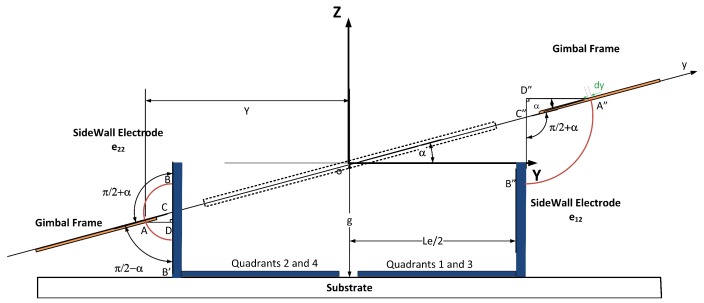
The projection of the mirror plate and the gimbal and sidewall electrodes on the YZ plane.

Now, we consider the effect of electrostatic forces from the sidewall electrodes on the gimbal frame, which also contribute to the rotation of the mirror. The gimbal frame does not rotate about the *Y*-axis, as shown in Figure 4, and it can only rotate about the *X*-axis. That means the electrostatic forces generated between sidewalls and gimbal frame changes the *α* angle only. Figure 4 shows the projection of the mirror plate and the gimbal frame on a plane parallel to YZ plane. Because of the larger width of the gimbal frame in the *y* direction, only the electrostatic torques and forces caused by Sidewalls 12, 22, 32 and 42, as labeled in Figure 1a, are considered in the simulations. The resulting electrostatic force in the *Z* direction and torque around the *X*-axis caused by Sidewall 12 forces on the top surface of the gimbal can be written:

(15) $$\begin{array}{c} Fz_{eg22t}=\frac{\varepsilon_{0}\cdot \sin^{2}\left(\pi /2-\alpha \right)\cdot V_{2}^{2}}{2{\left(\pi /2+\alpha \right)}^{2}}\int_{y_{5}}^{y_{6}}\int_{x_{5}}^{x_{6}}{\left\{\frac{1}{y\cos\alpha -\frac{L_{e}}{2}-t_{s}}\right\}}^{2}\;dx\;dy\\ Tx_{eg22t}=\frac{\varepsilon_{0}\cdot \sin^{2}\left(\pi /2-\alpha \right)\cdot V_{2}^{2}}{2{\left(\pi /2+\alpha \right)}^{2}}\int_{y_{5}}^{y_{6}}\int_{x_{5}}^{x_{6}}{\left\{\frac{1}{y\cos\alpha -\frac{L_{e}}{2}-t_{s}}\right\}}^{2}\cdot y\;dx\;dy \end{array}$$

where $t_{s}$ is sidewall thickness. Sidewall 22 also exerts electrostatic forces on the bottom surface of the gimbal frame. The corresponding electrostatic force and torque are:

(16) $$\begin{array}{c} Fz_{eg22b}=-\frac{\varepsilon_{0}\cdot \sin^{2}\left(\pi /2-\alpha \right)\cdot V_{2}^{2}}{2{\left(\pi /2-\alpha \right)}^{2}}\int_{y_{7}}^{y_{8}}\int_{x_{7}}^{x_{8}}{\left\{\frac{1}{y\cos\alpha -\frac{L_{e}}{2}-t_{s}}\right\}}^{2}\;dx\;dy\\ Tx_{eg22b}=\frac{\varepsilon_{0}\cdot \sin^{2}\left(\pi /2-\alpha \right)\cdot V_{2}^{2}}{2{\left(\pi /2-\alpha \right)}^{2}}\int_{y_{7}}^{y_{8}}\int_{x_{7}}^{x_{8}}{\left\{\frac{1}{y\cos\alpha -\frac{L_{e}}{2}-t_{s}}\right\}}^{2}\cdot y\;dx\;dy \end{array}$$

Similarly, the electrostatic force and torque caused by Sidewall 12 on the gimbal frame are:

(17) $$\begin{array}{cc} Fz_{eg12} & =-\frac{\varepsilon_{0}\cdot \sin^{2}\left(\pi /2-\alpha \right)\cdot V_{1}^{2}}{2{\left(\pi /2+\alpha \right)}^{2}}\int_{y_{9}}^{y_{10}}\int_{x_{9}}^{x_{10}}{\left\{\frac{1}{y\cos\alpha -\frac{L_{e}}{2}-t_{s}}\right\}}^{2}\;dx\;dy\\ Tx_{eg12} & =-\frac{\varepsilon_{0}\cdot \sin^{2}\left(\pi /2-\alpha \right)\cdot V_{1}^{2}}{2{\left(\pi /2+\alpha \right)}^{2}}\int_{y_{9}}^{y_{10}}\int_{x_{9}}^{x_{10}}{\left\{\frac{1}{y\cos\alpha -\frac{L_{e}}{2}-t_{s}}\right\}}^{2}\cdot y\;dx\;dy \end{array}$$

The electrostatic forces and torques caused by bottom electrodes was also added to the simulations.

##### Mechanical Spring Force

When the micro-mirror is actuated by sidewall electrodes, the electrostatic forces and torques will be balanced by mechanical restoring forces and torques.

(18) $$\begin{array}{cc} K_{x}\cdot \alpha & =2\cdot \left(Tx_{e22t}+Tx_{e22b}+Tx_{e12}+\left(Tx_{eg22t}+Tx_{eg22b}\right)+Tx_{eg12}+\sum_{m=1}^{2}Tx_{ebm}\right)\\ K_{y}\cdot \beta & =2\cdot \left(Ty_{e13t}+Tx_{e13b}+Ty_{e33}+Ty_{eb1}+Ty_{eb3}\right)\\ K_{z}\cdot z & =2\cdot (Fz_{e12}+Fz_{e22b}+Fz_{e22t}+Fz_{ge12}+Fz_{ge22b}+Fz_{ge22t}+Fz_{eb1a}+Fz_{eb2a}+\\ & \;Fz_{e33}+Fz_{e13b}+Fz_{e13t}+Fz_{eb1b}+Fz_{eb3b}) \end{array}$$

The micro-mirror model has a serpentine torsion bar with the spring constant equation [19] of:

(19) $$\begin{array}{c} \begin{array}{cc} K_{x} & =Ky={\left(\frac{l_{f}+\frac{6}{4}l_{p}+\frac{2l_{i}}{4}}{G.J_{r}}+\frac{2l_{0}}{EI}\right)}^{-1},\;K_{z}=\left(\frac{A}{B-C}\right)\\ A & =\frac{4l_{f}+6l_{p}+2l_{i}}{4EI}+\frac{l_{p}+7l_{0}}{4GJ_{r}},\;B=A\cdot \left(\frac{B_{1}}{4EI}+\frac{B_{2}}{4GJ_{r}}\right)\\ B_{1} & =0.3l_{f}^{3}+42.6l_{0}^{3}+4l_{f}^{2}l_{p}-4l_{f}l_{p}^{2}+2l_{p}^{3}+{\left(l_{f}-l_{p}\right)}^{2}\left(l_{p}+l_{i}\right)+\left(l_{f}-l_{p}\right)\left(l_{p}^{2}-l_{i}^{2}\right)+0.3l_{i}^{3}\\ B_{2} & =4l_{f}^{2}l_{0}+28l_{0}^{2}l_{p}+4{\left(l_{f}-l_{p}\right)}^{2}l_{0}+32l_{0}^{2}l_{i}\\ C & =\left(\frac{2l_{f}^{2}+6l_{f}l_{p}-2l_{p}^{2}+2l_{f}l_{i}-2l_{p}l_{i}+l_{i}^{2}}{4EI}+\frac{l_{f}l_{p}+7l_{f}l_{0}-4l_{p}l_{0}}{4GJ_{r}}\right)\cdot \left(\frac{0.5l_{f}^{2}+6l_{f}l_{p}+2l_{f}l_{i}-2l_{p}l_{i}-3l_{p}^{2}+l_{i}^{2}}{4EI}+\frac{8l_{f}l_{0}-2l_{p}l_{0}}{4GJ_{r}}\right) \end{array} \end{array}$$

where $K_{x}$, $K_{y}$ are the rotational stiffness of corresponding axis and $K_{z}$ is the translational stiffness of the serpentine spring. For *α* and *β* rotation angles, the polar moment of inertia ($J_{r}$) and the cross-section area moment of inertia (*I*) are [22]:

(20) $$\begin{array}{cc} J_{r} & =\frac{t_{b}^{3}\cdot w}{3}\cdot \left(1-\frac{192t_{b}}{w\cdot \pi^{5}}\cdot \sum_{i=1,3,5,\cdots }\frac{1}{i^{5}}\cdot \tanh(\frac{i\pi \cdot w}{2t_{b}})\right)\\ I & =\frac{t_{b}^{3}\cdot w}{12} \end{array}$$

where i=1 was used in the series expansion. Furthermore, in Equation (19), $t_{b}$ and *w* are the thickness and width of the serpentine spring, respectively given in Figure 2. The mirror dimensions are provided in [19].

### 2.3. Static Simulation Results

**Figure 5:**
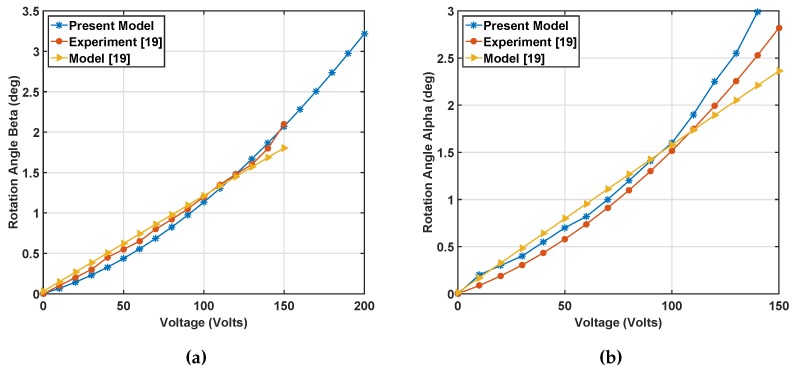

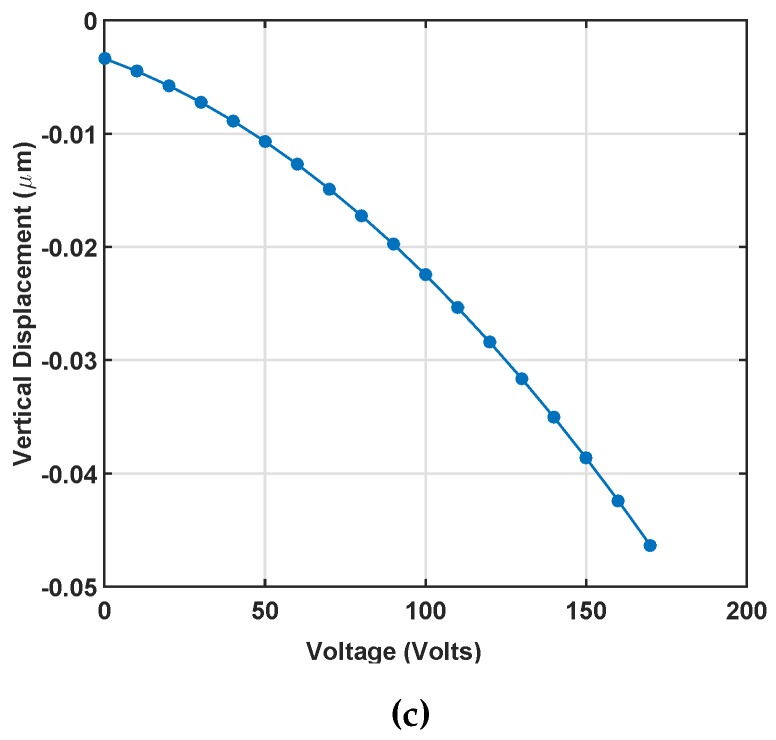
(**a**) Static simulation of rotation angle *β* *versus* differential voltage *V_diff_* when *V_bias_* = 55 V; (**b**) static simulation of rotation angle *α versus* differential voltage *V_diff_* *versus* differential voltage *V_diff_* when *V_bias_* = 55 V; (results presented by yellow and red colors are adapted from [19]) (**c**) vertical displacement of the mirror when *V_bias_* = 55 V obtained using the present model.

For obtaining the rotation angles, the summation of all of the torques in corresponding directions of *x* and *y* are found considering the voltages of each quadrant. Equation (18) was solved at different differential voltages, as indicated in Equations (1) and (3). In the simulations, the fringe field effect from the mirror thickness is considered negligible. Figure 5a,b shows the rotation angles *β* and *α* *versus* differential voltage when bias voltage $V_{bias}=55\;V$ and $V_{diff}$ ranges from 0–150 V, and Figure 5c shows the vertical displacement of the center of the mirror. There was no experimental data to compare, but as can be seen, the vertical displacement is negligible. In Figure 5a,b, the present model can predict the nonlinear trend in the experimental static response with a close agreement, while the previous model [19] reveals a linear trend and diverges from the experiment beyond 1.7 degrees. The close prediction of nonlinear response in our model is from considering the electrostatic forces on the mirror’s top surface when the angles increase, which was not included in the prior work [19]. The accuracy in predicting static nonlinearity helped us in the simulation of nonlinear dynamic response (Section 3), which has not been reported previously. It should be noted that for *α* angle simulations, we used the nominal dimensions, as listed in Figure 2, for calculating serpentine stiffness. For the angle beta, we used the effective serpentine dimensions of $l_{f}=135\;\mu$m, $l_{p}=120\;\mu$m, $l_{i}=120\;\mu$m, $t_{b}=9\;\mu$m and $l_{0}=10\;\mu$m to account for the difference in the spring stiffness about the two axes. Same dimensions are used for dynamic simulations.

## 3. Dynamic Simulation

### 3.1. Equations of Motion

In this section, we investigate the dynamic behavior of the mirror for different excitation voltages. Our mirror is excited by AC and DC combined loads. $V_{bias}$ in static simulation is replaced by $V_{DC}$ voltage. The governing equation of motion can be obtained using Lagrange’s equation. Lagrange’s equation may be written:

(21) $$\frac{d}{dt}\left(\frac{\partial T}{\partial \dot{q_{i}}}\right)-\frac{\partial T}{\partial q_{i}}+\frac{\partial V}{\partial q_{i}}+\frac{\partial D}{\partial \dot{q_{i}}}=\Gamma_{i}$$

In this equation, $q_{i}$ is a generalized coordinate, *V* is the potential energy, *T* is kinetic energy, *D* is the Rayleigh dissipation function that depends on viscous damping and Γ is the torque applied to the system from electrostatic actuation. The rotational motion of the mirror is described by two generalized coordinates of *α* and *β*. The potential energy of the mirror is given by Equation (22):

(22) $$V=\frac{1}{2}K_{x}\alpha^{2}+\frac{1}{2}K_{y}\beta^{2}+\frac{1}{2}K_{z}z^{2}$$

where $K_{x}$ and $K_{y}$ are the torsional stiffness of serpentine for *α* and *β*. Furthermore, $K_{z}$ is the bending stiffness of the serpentine for vertical motion. The change in height of the center of mass is significantly small, so the potential energy of the mirror plate and gimbal frame can be neglected. The total kinetic energy equation is given as:

(23) $$T=\frac{1}{2}\left(\left(J_{p}+J_{g}\right)\cdot {\dot{\alpha }}^{2}+J_{p}\cdot {\dot{\beta }}^{2}+\left(m_{p}+m_{g}\right)\cdot {\dot{z}}^{2}\right)$$

(24) $$\begin{array}{cc} m_{p} & =\rho \cdot l_{m}^{2}\cdot t_{m}\\ m_{g} & =\rho \cdot t_{g}\cdot \left(L_{gw}\cdot L_{gl}-L_{gwi}\cdot L_{gi}\right)\\ J_{p} & =m_{p}\cdot \left(\frac{l_{m}^{2}+t_{m}^{2}}{12}\right)\\ J_{g} & =m_{g}\cdot \left(\frac{L_{gl}^{2}+t_{g}^{2}}{12}-\frac{L_{gi}^{2}+t_{g}^{2}}{12}\right)=m_{g}\cdot \left(\frac{L_{gl}^{2}-L_{gi}^{2}}{12}\right) \end{array}$$

where *ρ* is the density of mirror material, $J_{p}$ and $J_{g}$ are the mass moment inertia of the mirror plate and gimbal, $l_{m}$ and $t_{m}$ are length and thickness of the mirror plate, respectively, $m_{p}$ and $m_{g}$ are the masses of the plate and gimbal frame, respectively, and $L_{gw}$, $L_{gl}$, $L_{gwi}$, $L_{gi}$ and $t_{g}$ are the mirror outer width, gimbal outer length, mirror inner width, gimbal inner length and thickness of gimbal frame, respectively. The last parameter for Lagrange Equation (21) is energy dissipation.

(25) $$\begin{array}{c} D=\frac{1}{2}\left(d_{1}\cdot {\dot{\alpha }}^{2}+d_{2}\cdot {\dot{\beta }}^{2}+d_{2}\cdot {\dot{z}}^{2}\right) \end{array}$$

where $d_{1}$ and $d_{2}$ represent damping coefficients for rotations about *x* and *y*, respectively. Substituting the relevant terms into Equation (21), we obtain the equations of motion for *α* and *β*:

(26) $$\begin{array}{cc} \ddot{\alpha }+2\zeta_{1}\omega_{\alpha }\cdot \dot{\alpha }+\omega_{\alpha }^{2}\cdot \alpha & =\frac{\sum \Gamma_{\alpha }}{\left(J_{p}+J_{g}\right)}\\ \ddot{\beta }+2\zeta_{2}\omega_{\beta }\cdot \dot{\beta }+\omega_{\beta }^{2}\cdot \beta & =\frac{\sum \Gamma_{\beta }}{J_{p}}\\ \ddot{z}+2\zeta_{2}\omega_{z}\cdot \dot{z}+\omega_{z}^{2}\cdot z & =\frac{\sum F_{z}}{m_{p}+m_{g}} \end{array}$$

where $2\zeta_{1}\omega_{\alpha }=\frac{d_{1}}{J_{p}+J_{g}}$, $2\zeta_{2}\omega_{\beta }=\frac{d_{2}}{J_{p}}$, $2\zeta_{2}\omega_{z}=\frac{d_{2}}{m_{p}+m_{g}}$ and natural frequencies about the *X*-, *Y*- and *Z*-axis are $\omega_{\alpha }^{2}$=$\frac{K_{x}}{J_{p}+J_{g}}$, $\omega_{\beta }^{2}$=$\frac{K_{y}}{J_{p}}$, $\omega_{z}^{2}$=$\frac{K_{z}}{m_{p}+m_{g}}$. The state space equation can be written:

(27) $$\begin{array}{cc} x_{1}^{{}^{\prime }} & =x_{2}\\ x_{2}^{{}^{\prime }} & =-2\zeta_{1}\omega_{\alpha }.x_{2}-\omega_{\alpha }^{2}.x_{1}+\frac{\sum \Gamma_{\alpha }}{J_{p}+J_{g}}\\ x_{3}^{{}^{\prime }} & =x_{4}\\ x_{4}^{{}^{\prime }} & =-2\zeta_{2}\omega_{\beta }.x_{4}-\omega_{\beta }^{2}.x_{3}+\frac{\sum \Gamma_{\beta }}{J_{p}}\\ x_{5}^{{}^{\prime }} & =x_{6}\\ x_{6}^{{}^{\prime }} & =-2\zeta_{3}\omega_{z}.x_{6}-\omega_{z}^{2}.x_{5}+\frac{\sum F_{z}}{m_{p}+m_{g}} \end{array}$$

### 3.2. Dynamic Simulation Results

**Figure 6:**
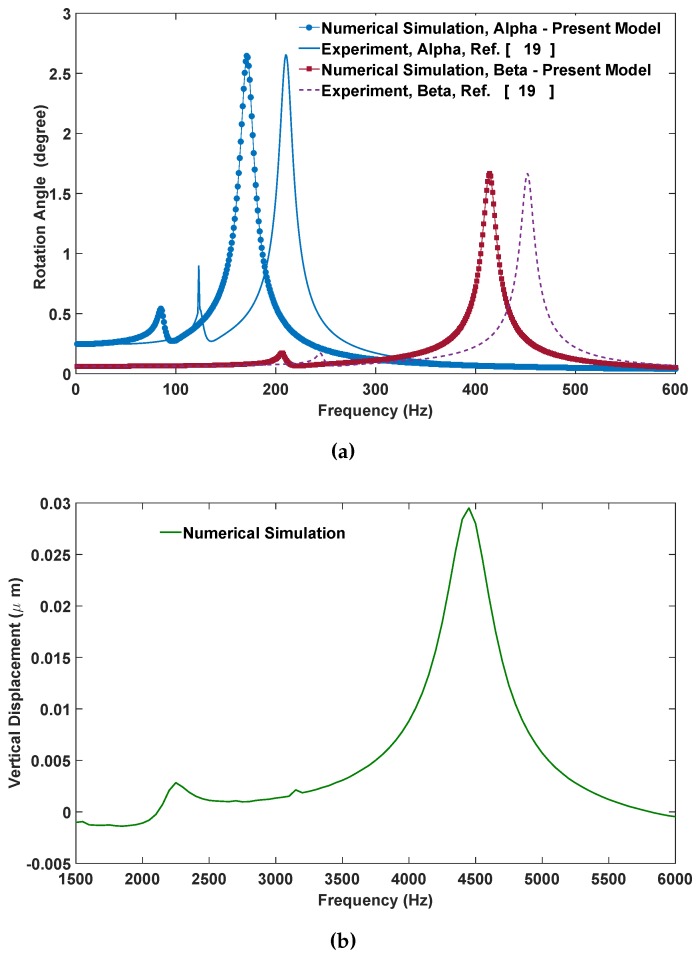
(**a**) Experimental and numerical comparison for the frequency response of angle alpha when $V_{DC}=\;55\;V$ and $V_{ac}=25\;V$; experimental and numerical comparison for frequency response of the angle beta when $V_{DC}=55\;V$ and $V_{ac}=15\;V$; (results presnted by solid blue and dash red lines are adpated from [19]) (**b**) the frequency response of the mirror for vertical displacement is numerically simulated when $V_{DC}=55\;V$ and $V_{ac}=25\;V$.

The transient response and frequency response of the dynamic behavior were simulated using the Runge–Kutta numerical integration method assuming zero initial conditions. Comparing to the experimental data from [19], we estimated the damping ratios as $\left[\zeta_{1};\;\zeta_{2};\;\zeta_{3}\right]=\left[0.0382;\;0.0208;\;0.0208\right]$ for *α* and *β* scanning angles, respectively. The damping ratios about two axes of rotations are different, as the corresponding resonance frequencies are not the same. Furthermore, note that because of the geometrical constraints, the maximum reachable *α* angle is around 19 degrees and the *β* angle is around 35 degrees, since the gimbal and the mirror plate hit the substrate, respectively. The simulations presented below are within these maximum angle ranges. In Figure 6a, the frequency responses of the scanning angles at $V_{bias}$= 55 V are presented. Figure 6b demonstrates the frequency response of the vertical displacement at $V_{bias}$= 55 V. The numerical simulation for scanning angles shows good accuracy with the experimental results. These figures also show that for both the *α* and *β* angles, superharmonic resonances at orders of two are observed, as reported in the experimental data. It should be noted that secondary resonances have not been reported for the mirror with sidewall electrodes in the literature. There is a slight difference between experiments and simulation that can be explained from the deviation of nominal dimensions after fabrication. The authors did not have access to the actual device to measure the exact dimensions under an optical profiler to examine the variation of thickness across the mirror plate or the gimbal frame. These variations are responsible for the mismatch of the resonant frequencies.

### 3.3. Analytical Explanation of Secondary Resonances

Close agreement between experimental results and dynamic simulation confirmed accurate modeling of the electrostatic field for the rotational micro-mirror. At low voltages, superharmonic resonance showed the stiffness nonlinearity in the system. In this section, we examine the effect of increasing forcing and decreasing damping on primary and secondary resonances, then describe the underlying nonlinearities from driven mathematical equations of motion. The primary resonance happens at the frequency close to the natural frequency about each axis of rotation, and secondary resonance, such as superharmonic resonance, appears at a frequency away from the natural frequency when nonlinear stiffness terms are present [23,24]. Secondary resonances arise from nonlinear coupling, restoring force that is caused by the electrostatic force on the mirror. The secondary resonances are activated at the high forcing and low damping (low pressure environment).

The cubic stiffness nonlinearity causes the subharmonic resonance of order 1/3 and superharmonic resonance of order three. On the other hand, quadratic nonlinearity triggers subharmonic resonance of order 1/2 and superharmonic resonance of order two. Subharmonic resonances are secondary resonances as a result of nonlinear spring forces, which generate large responses at a fraction of the excitation frequency. For instance, when the excitation frequency is NΩ, the system responds at Ω, where Ω is the natural frequency of the system. This means that this system has subharmonic resonances at the order of 1/N, where *N* is an integer greater than zero.

**Figure 7:**
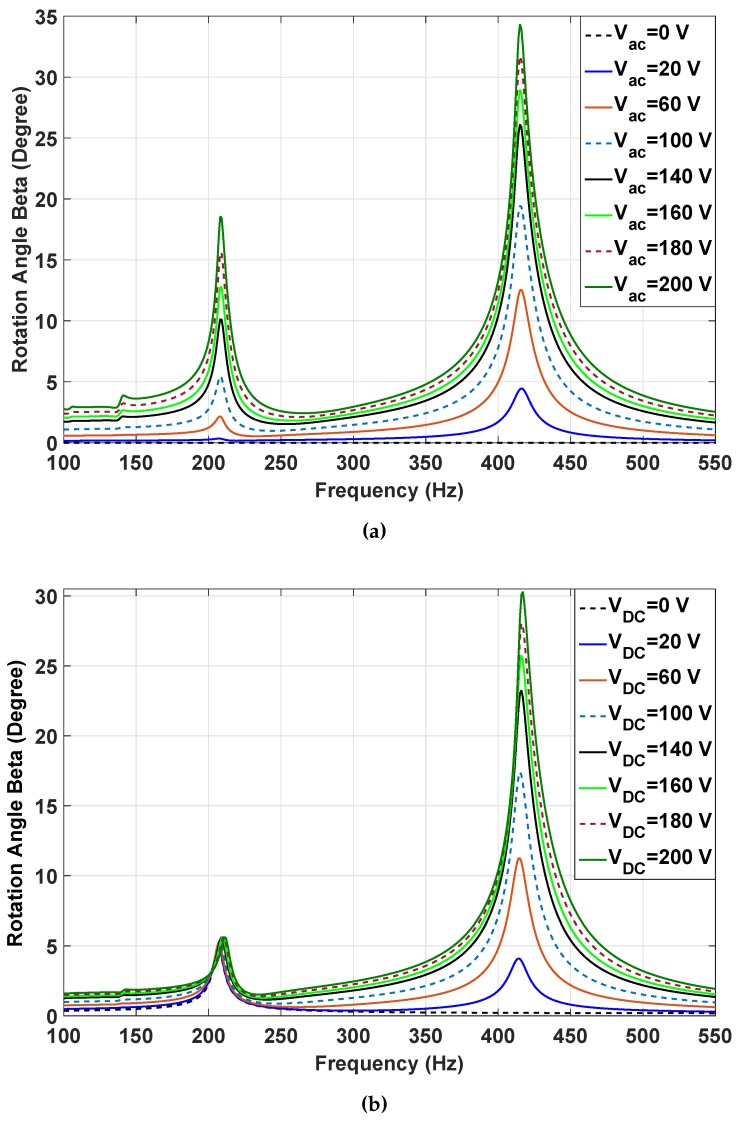
(**a**) The secondary resonance softening behavior of the beta angle when $V_{DC}=115\;V$ and $V_{ac}$ changes from 0 to 200 V; (**b**) the secondary resonance of beta angle when $V_{ac}=100\;V$ and $V_{DC}$ changes from 0 to 200 V.

Superharmonic resonances are large responses at integer multiples of the excitation frequency. For instance, when the excitation frequency is $\frac{\Omega }{N}$, the system responds at Ω, where Ω is the natural frequency of the system. In this case, we deduce that the system has superharmonic resonance of order *N*. Figure 7a,b shows the frequency response as the AC and DC voltages are increased, respectively. The figures reveal the appearance of primary resonance (around 420 Hz) and two superharmonic resonances of order two (around 210 Hz) and order three (around 140 Hz), which increase with the increasing the voltage. One can deduce that by increasing the voltage, the primary resonance peak inclines to the left (softening), as in Figure 7a,b. We expect that the frequency peak could considerably bend to the left with a further increase of voltage. However, because of the physical limitation on the rotation angle (35 degrees), a further increase of the voltage was not meaningful. It is noted that the effect of AC voltage on the superharmonic resonance of order two was more prominent than that of the DC voltage. The results indicate that there are nonlinear stiffness terms in the system with a dominant quadratic nonlinearity, as the superharmonic resonance of order two is the prominent secondary resonance.

To describe the quadratic nonlinearity in the system, which is the cause of dominant secondary superharmonic resonance seen in the system, we scrutinize the mathematical equation of motion of the mirror, looking for nonlinear stiffness terms. We start from the torque about the *Y*-axis $Ty_{e13t}$ (Equation (11)):

(28) $$Ty_{e13t}=-\frac{\varepsilon_{0}\cdot \sin^{2}\left(\pi /2-\beta \right)\cdot V_{1}^{2}}{2{\left(\pi /2-\beta \right)}^{2}}\cdot \int_{y_{13}}^{y_{14}}\int_{x_{13}}^{x_{14}}{\left\{\frac{1}{\frac{L_{e}}{2}-x\cdot \cos\beta }\right\}}^{2}\cdot x\;dx\;dy$$

which can be integrated analytically to yield:

(29) $$\begin{array}{c} Ty_{e13t}=-\frac{\varepsilon_{0}\cdot \sin^{2}\left(\pi /2-\beta \right)\cdot V_{1}^{2}}{2{\left(\pi /2-\beta \right)}^{2}}\left(y_{14}-y_{13}\right)\cdot \left(\frac{\frac{x_{14}}{\frac{Le}{2}-x_{14}.\cos\left(\beta \right)}-\frac{x_{13}}{\frac{Le}{2}-x_{13}\cdot \cos\left(\beta \right)}}{\cos(\beta )}-\frac{\ln\left(\frac{\frac{Le}{2}-x_{14}\cdot \cos\left(\beta \right)}{\frac{Le}{2}-x_{13}\cdot \cos\left(\beta \right)}\right)}{\cos{\left(\beta \right)}^{2}}\right) \end{array}$$

A similar analysis can be applied to other torque equation. In Equation (29), $y_{14}$, $y_{13}$, $x_{14}$, $x_{13}$ are constants, and the only variable is *β*. Knowing the Taylor expansion around zero to be:

(30) $$\begin{array}{c} f\left(\beta \right)=f\left(0\right)+\frac{f^{\prime }\left(0\right).\left(\beta -0\right)}{1!}+\frac{f^{\prime \prime }\left(0\right).{\left(\beta -0\right)}^{2}}{2!}+\frac{f^{\prime \prime \prime }\left(0\right).{\left(\beta -0\right)}^{3}}{3!}+\cdots +\frac{f^{n}\left(0\right)\cdot {\left(\beta -0\right)}^{n}}{n!}=\sum_{n=1}^{\infty }\frac{f^{n}\left(0\right)}{n!}{\left(\beta -0\right)}^{n} \end{array}$$

We write a Taylor series expansion up to order three for f(β):

(31) $$f\left(\beta \right)=\ln\left(\frac{\frac{Le}{2}-x_{14}\cdot \cos\left(\beta \right)}{\frac{Le}{2}-x_{13}\cdot \cos\left(\beta \right)}\right)$$

The expansion yields:

(32) $$\begin{array}{cc} f(\beta ) & =\left(\sigma_{2}\sin\left(\beta \right)-\sigma_{1}\sin\left(\beta \right)\right)\cdot \beta +\left(\sin{\left(\beta \right)}^{2}\cdot \left(\sigma_{1}^{2}-\sigma_{2}^{2}\right)+\cos\left(\beta \right)\cdot \left(\sigma_{2}-\sigma_{1}\right)\right)\cdot \beta^{2}\\ & +\left(2\sin{\left(\beta \right)}^{3}\left(\sigma_{2}^{3}-\sigma_{1}^{3}\right)+3\cos\left(\beta \right)\sin\left(\beta \right)\left(\sigma_{1}^{2}-\sigma_{2}^{2}\right)+\sin\left(\beta \right)\sigma_{1}\right)\cdot \beta^{3} \end{array}$$

(33) $$\begin{array}{c} \sigma_{1}=\frac{y_{13}}{\frac{Le}{2}-y_{13}.\cos\left(\beta \right)},\;\sigma_{2}=\frac{y_{14}}{\frac{Le}{2}-y_{14}.\cos\left(\beta \right)},\;a_{0}=\left(y_{14}-y_{13}\right)\left(\frac{2x_{14}}{Le-2x_{14}}-\frac{2x_{13}}{Le-2x_{13}}\right) \end{array}$$

For small rotation angles, sin(β)≈0, cos(β)≈1 and $\frac{\varepsilon_{0}\cdot \sin^{2}\left(\pi /2-\beta \right)\cdot V_{1}^{2}}{2{\left(\pi /2-\beta \right)}^{2}}\approx C\cdot V_{1}^{2}$, where *C* is a constant value. Using the appropriate terms, odd power terms that vanish in Equations (32) and (29) can be written as:

(34) $$Ty_{e13t}=-CV_{1}^{2}a_{0}\left(1-\beta^{2}\right)$$

$a_{0}$ is defined in Equation (33). Consequently, the equation of motion for *β* is obtained as:

(35) $$J_{p}\cdot \ddot{\beta }+K_{t2}\cdot \beta -r_{1}\cdot \beta^{2}\cdot V^{2}=-r_{2}\cdot V^{2}$$

where $r_{1}$ and $r_{2}$ are constants.

The simplified equation of motion reveals the fact that excitation voltage changes the stiffness of the system in linear and nonlinear fashions, which clearly describes softening and superharmonic resonances in the system. The third term in Equation (35) shows that the dominant stiffness nonlinearity in the system is quadratic, which explains the measured superharmonic resonance of order two, even at small rotation angles, as in Figure 6a. We can also see the slight softening caused by quadratic nonlinearity in Figure 7a,b. The quadratic stiffness nonlinearity does not refer to the mechanical structure here, but indicates the electromechanical coupling effect caused by the electrostatic torque (Equation (34)). As the angles becomes larger, the odd terms in the Taylor series expansion become considerable, meaning that at larger voltages, cubic nonlinearity arises and is responsible for superharmonic resonances of order three in Figure 7a.

**Figure 8:**
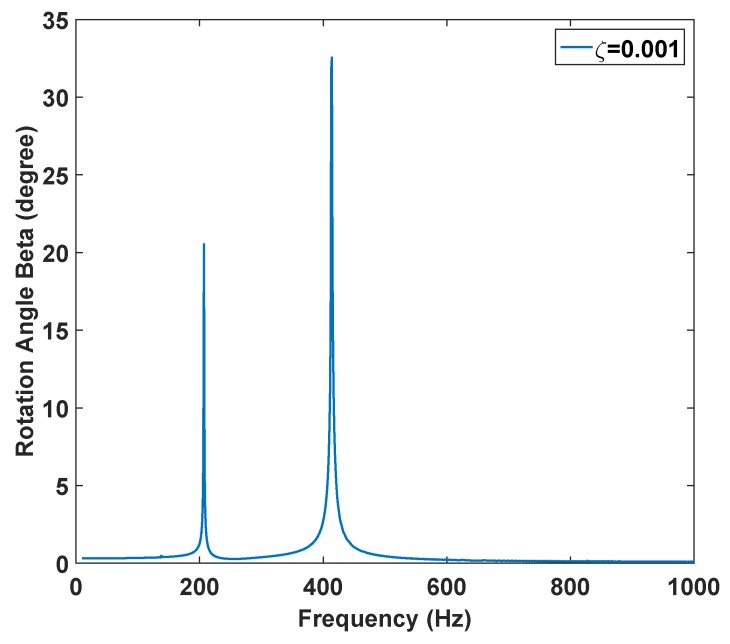
The effect of the decreased damping ratio on the nonlinear dynamic behavior of the beta angle when $V_{DC}=45\;V$ and $V_{ac}=65\;V$ for $\zeta_{2}=0.001$.

As most of the MEMS mirrors are vacuum packaged, they experience a reduced pressure environment. Damping ratios are lower at reduced pressure values. The effect of the decrease of the damping ratio on the frequency response is analogous to the increase of forcing. In Figure 8, we examined the effect of a reduced damping ratio, 0.001 compared to 0.0208 in Figure 7a,b, on the frequency response. As is observed, the superharmonic resonance of order two is the dominant secondary resonances and becomes more significant at smaller damping ratios (reduced pressure environment).

**Figure 9:**
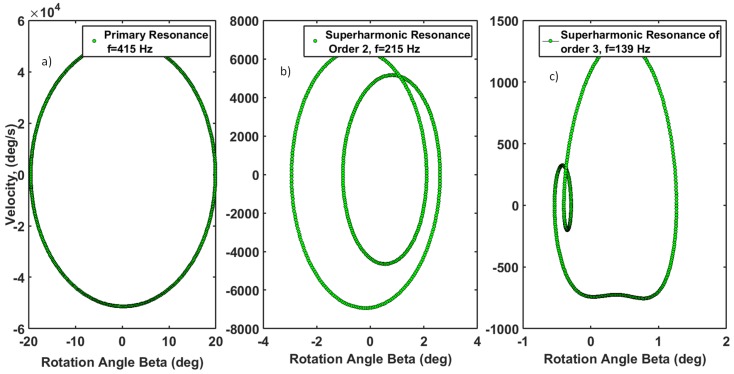
Phase portraits of the beta angle for $V_{ac}=100\;V$, $V_{DC}=115\;V$ and $\zeta_{2}=0.0208$. (**a**) Primary resonance. These phase portraits are plotted for initial values of x(0)=0, $\dot{x}=0$. (**b**) Superharmonic resonance of order two. (**c**) Superharmonic resonance of order three.

Evolution of phase portraits at primary and secondary resonances of the *β* angle are shown in Figure 9. Figure 9a shows how the elliptic trajectory of the primary resonance is converted to multiple enclosed ellipses, as in the case of superharmonic resonance of order two in Figure 9b. Figure 9c shows a superharmonic resonance of order three. As can be deduced, the trajectories shrink along the angular position and angular velocity axes as the order of resonance increases. That indicates a higher signal to noise ratio at a lower order of resonance.

## 4. Conclusions

Mathematical modeling and dynamic simulation of a bi-axial MEMS mirror with sidewall and bottom electrodes are presented here that are in close agreement with the reported experimental data. We account for the increase of electrostatic field as the rotation angle increases and are able to predict the nonlinear dynamic behavior. The analytical model describes softening behavior and nonlinear superharmonic resonances observed in the experiment. The electrostatic force causes an electromechanical coupling effect that acts as a quadratic stiffness term responsible for frequency softening the superharmonic resonance of order two. The effect of damping on the frequency response of the mirror is examined, which revealed that superharmonic resonances become more significant. The presented performance analysis of the mirror with sidewall electrodes is valuable for predicting the mirror behavior in laser steering applications.

## Appendix: Integral Boundaries

(A1) $$\begin{array}{c} \begin{array}{cc} x_{1} & =\frac{w_{f}}{2}\;y_{1}=\frac{Le}{2\cos(\alpha_{0})}-\frac{Le}{2}\tan\left(\alpha_{0}\right)-\left(g-h\right)\\ x_{2} & =\frac{lm}{2}\;y_{2}=\frac{lm}{2}\\ x_{3} & =\frac{w_{f}}{2}\;y_{3}=\frac{Le}{2\cos(\alpha_{0})}-g+\frac{Le}{2}\tan\left(\alpha_{0}\right)-\left(g-h\right)\\ x_{4} & =\frac{lm}{2}\;y_{4}=\frac{lm}{2}\\ x_{5} & =\frac{w_{f}}{2}\;y_{5}=\frac{Le}{2\cos(\alpha_{0})}-g+\frac{Le}{2}\tan\left(\alpha_{0}\right)-\left(g-h\right)\\ x_{6} & =\frac{Le}{2}+t_{s}\;y_{6}=\frac{Lgl}{2\cos(\alpha_{0})}+\frac{Le}{2}\tan\left(\alpha_{0}\right)\\ x_{7} & =\frac{w_{f}}{2}\;y_{7}=\frac{Lgl}{2}-g_{wy}\\ x_{8} & =\frac{Le}{2}+t_{s}\;y_{8}=\frac{Lgl}{2\cos(\alpha_{0})}+\frac{Le}{2}\tan\left(\alpha_{0}\right)\\ x_{9} & =\frac{w_{f}}{2}\;y_{9}=\frac{Lgl}{2}-g_{wy}\\ x_{10} & =\frac{Le}{2}+t_{s}\;\;y_{10}=\frac{Lgl}{2\cos(\alpha_{0})}-\frac{Le}{2}\tan\left(\alpha_{0}\right)+2g-h \end{array} \end{array}$$
